# Lipase-Catalyzed Synthesis of Renewable Plant Oil-Based Polyamides

**DOI:** 10.3390/polym11111730

**Published:** 2019-10-23

**Authors:** Maja Finnveden, Peter Hendil-Forssell, Mauro Claudino, Mats Johansson, Mats Martinelle

**Affiliations:** 1 Department of Industrial Biotechnology, KTH Royal Institute of Technology, SE-106 91 Stockholm, Sweden; majafi@kth.se (M.F.);; 2 Department of Fibre and Polymer Technology, KTH Royal Institute of Technology, SE-100 44 Stockholm, Sweden; maurojcclaudino@gmail.com (M.C.); matskg@kth.se (M.J.)

**Keywords:** *Candida antarctica* lipase B, bio-based polyamides, enzymatic polymerization

## Abstract

Enzyme catalyzed synthesis of renewable polyamides was investigated using *Candida antarctica* lipase B. A fatty acid-derived AB-type functional monomer, having one amine and one methyl ester functionality, was homopolymerized at 80 and 140 °C. Additionally, the organobase 1,5,7-triazabicyclo[4.4.0]dec-5-ene (TBD) was used as a catalyst. The results from the two catalysts were comparable. However, the amount of lipase added was 1.2 × 10^3^ times lower, showing that the lipase was a more efficient catalyst for this system as compared to TBD. Moreover, the AB-type monomer was copolymerized with 1,12-diaminododecane to synthesize oligoamides of two different lengths.

## 1. Introduction

In modern chemistry, implementing sustainability is an important task. At the heart of the principles of green chemistry, both catalysis and the use of renewable resources can be found [1,2]. Enzymatic polymerization has been shown to be a potential alternative to traditional chemical polymerization, e.g., lipases are commonly employed for polycondensations, see recent reviews [3,4,5,6]. The first lipase-catalyzed polycondensations were reported in the early 1980s [7,8] and lipases have since then been established as important synthesis tools for polymeric materials. Amongst the benefits of enzymatic polymerization are: mild reaction conditions, few by-products, and high catalytic activity. Although lipases can catalyze the formation of amide bonds, and polyamides are an important group of polymers, most work reported on lipase-catalyzed polymerization has been on polyester synthesis [6]. There are few reports in the literature on the use of enzymatic routes for the synthesis of bio-based polyamides [9,10,11,12,13,14]. One reason for the lack of reports on the lipase-catalyzed polyamide synthesis might be that polyamides solidify before they can reach high molecular weights because of hydrogen bonding effects. Polyamides have high melting temperatures (*T*_m_) (130–200 °C), which is true even for oligoamides. To circumvent the issue of high *T*_m_, previous enzyme catalyzed polyamide synthesis has typically been performed in two or three steps, increasing temperature and decreasing pressure with time to obtain homogeneous reaction mixtures, thus obtaining high molecular weights [9]. 

An interesting synthetic approach for the preparation of renewable monomers for polyamides via radical thiol–ene coupling of cysteamine to unsaturated fatty acid esters was presented by Türünç et al. [15]. The resulting AB-type, long and branched, monomers were homopolymerized using the base triazabicyclodecene (TBD) as a catalyst. TBD is a strong guanidine base that has previously been used to catalyze a variety of transformations including transesterification reactions [16] and polyamide synthesis [15,17]. The resulting polyamide products were still viscous at room temperature. Thus, we hypothesized that it should be possible to utilize lipase catalysis, without the oligomers solidifying. Among the lipases used in enzymatic polymerization, Novozym 435 (immobilized *Candida antarctica* lipase B (CalB)) is most widely used [18].

**Scheme 1:**
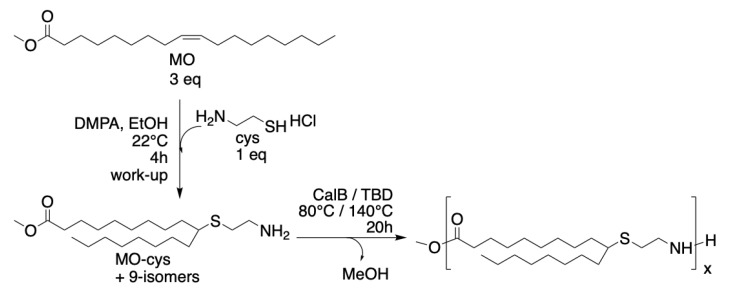
Reaction scheme for synthesis of MO-cys by thiol-ene addition of cysteamine to methyl oleate and homopolymerization of MO-cys using CalB or TBD.

**Scheme 2:**
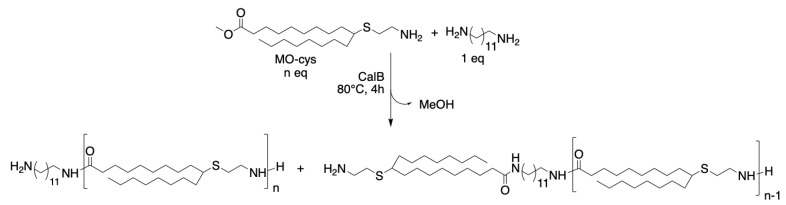
Reaction scheme of copolymerization of MO-cys and 1,12-diaminododecane catalyzed by CalB, where n = 2 or 4.

In this paper, we build on the original work by Türünç et al. [15] and compare the catalyst TBD with the lipase CalB, for the synthesis of renewable polyamides. The comparison of the catalysts was made for the homopolymerization of the renewable AB-type monomer at temperatures from 80 to 140 °C (Scheme 1). The mole% of CalB used was 1.2 × 10^3^ times less compared to TBD, however the obtained conversions were equivalent. Additionally, the lipase was used to synthesize amine end-capped oligoamides (Scheme 2). 

## 2. Results

Cysteamine (cys) was coupled to methyl oleate (MO) via thiol–ene addition to form the renewable AB-type methyl ester amine monomer (MO-cys). The method was first described by Türünç et al. [15] and the synthesis described herein was performed with some modifications, as shown in Scheme 1. The thiol–ene reaction is neither regioselective nor enantioselective and thus the addition of the cysteamine occurs both at the 9 and 10 position of the methyl oleate, yielding four isomers. The results from the synthesis of MO-cys agree with previous reports on thiol-ene coupling of 1,2-disubstituted alkene monomers [19,20].

**Figure 1:**
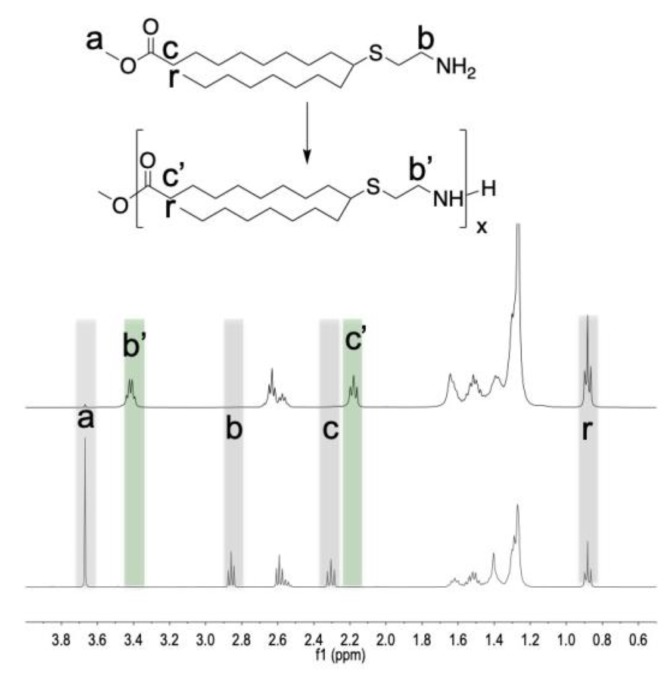
^1^H-NMR spectra of CalB-catalyzed homopolymerization of MO-cys at 140 °C, from 4.0–0.5 ppm (enlargements of the whole area). The bottom spectrum shows the start before the addition of CalB and the top spectrum was acquired after 3.5 h. Conversion was calculated from the formation of peak b’ using r as a reference. Signals highlighted in grey have been assigned to atoms present from the start (a, b, c and r) and signals highlighted in green have been assigned to atoms in the polymer (b’, c´ and r).

The homopolymerization of MO-cys was catalyzed by the enzyme *Candida antarctica* lipase B (CalB) and the organobase TBD in bulk at two different temperatures 80 or 140 °C (Scheme 1). The CalB-catalyzed homopolymerization of MO-cys at 140 °C was followed by ^1^H-NMR. The spectra at the start and after 3.5 h are shown in Figure 1. The formation of the polyamides was evidenced by ^1^H-NMR analysis showing a shift of the peak corresponding to the protons at the β-positions of the thioether at 2.9 ppm to 3.4 ppm when the amide was formed (15). Furthermore, the peak at 3.7 ppm, corresponding to the methyl ester of MO-cys decreased. The protons corresponding to the α-carbon of the carbonyl of MO-cys shifted from 2.3 ppm to 2.2 ppm. Table 1 (Entry 1–4) summarizes the results from the homopolymerizations of MO-cys catalyzed by CalB and TBD.

**Figure 2:**
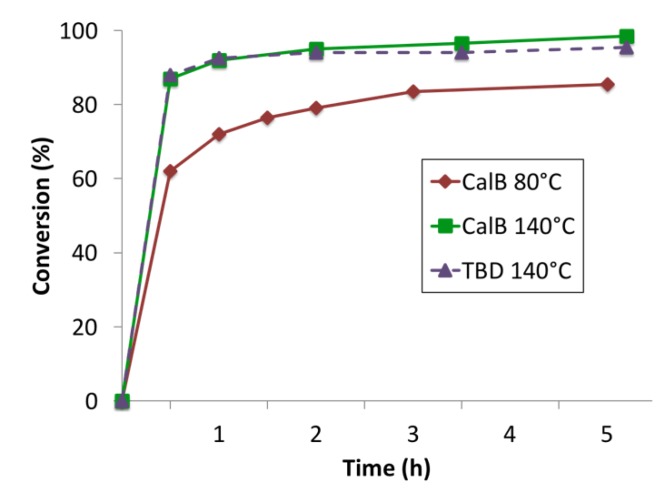
Amide formation in different catalytic systems, during homopolymerization of MO-cys over time (following the amide peak at 3.4 ppm by ^1^H-NMR).

As can be seen in Table 1, the homopolymerizations of MO-cys were performed for 20 h. However, after 5 h the reaction mixtures became so viscous that the magnetic stirring stopped. Consequently, the reactions showed little change in conversion after 5 h. Figure 2 shows the conversion to amide over the first 5 h of reactions catalyzed by CalB at 80 and 140 °C, and TBD at 140 °C. TBD was used as a catalyst at 80 °C, but only endpoint samples were withdrawn (results shown in Table 1).

At 140 °C, almost 90% conversion of MO-cys was observed after 30 minutes when CalB or TBD were used as catalysts (Figure 2). When the reaction was performed without catalyst, 30% conversion of the methyl ester was observed (Table 1). The methyl ester was converted to amide, evidenced by ^1^H-NMR analysis (formation of the signal assigned to the hydrogen atoms on the amide α-carbon). However, the reaction mixture turned black and additional side-reactions associated with the thioether were observed. 

At 80 °C, 70% conversion was reached within the first hour, as can be seen in Figure 2. After 20 h, around 85% conversion was reached. This can be related to the reaction without catalyst at 80 °C, which showed 20% conversion of the methyl ester attributed to amide formation after 20 h (Table 1). At 80 °C, no side-reactions were observed. The reason for the background amide formation without catalyst can be attributed to the amine. Although, methyl esters are not generally reactive towards amines, the two can react to form amides under conditions such as high temperatures [22].

It should be noted that a reaction temperature of 140 °C is considered a high temperature for utilizing CalB as a catalyst. Although, without CalB losing its activity completely during the reaction, temperatures of up to 150 °C have been reported to work well [9]. Nevertheless, the possibilities of reusing CalB after reactions run at such high temperatures are minimal [23,24]. Analysis by ^1^H-NMR and size-exclusion chromatography (SEC) (Supporting info Table S1) showed similar results for CalB and TBD at both temperatures.

**Figure 3:**
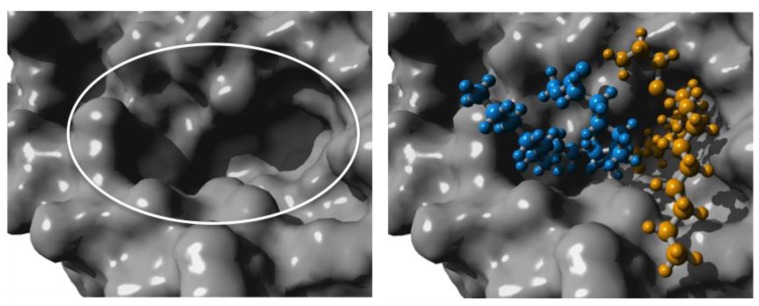
Close up of the surface around the active site of *Candida antarctica* lipase B. The white oval in the left frame marks the narrow binding pocket. In the frame to the right, two MO-cys molecules were modelled as a tetrahedral intermediate, into the active site. The orange MO-cys represents the acyl donor and the blue MO-cys the acyl acceptor. Figure made in Yet Another Scientific Artificial Reality Application (YASARA) based on PDB ID: 1LBT [25].

**Figure 4:**
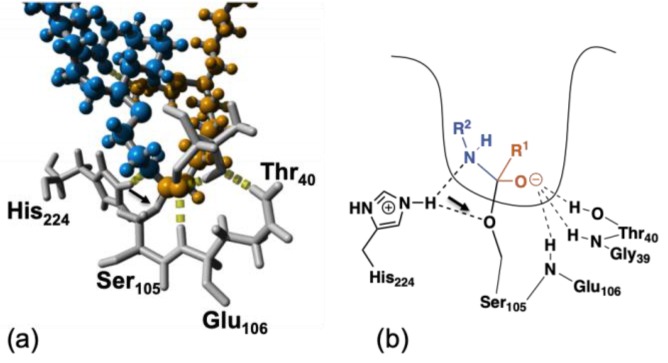
Close up of the active site (**a**) and schematic representations (**b**), where the orange MO-cys represents the acyl donor, the blue MO-cys represents the acyl acceptor, the important amino acids are shown in grey, and R^1^ and R^2^ denote the rest of the MO-cys. Figure made in YASARA and modelled based on PDB ID: 1LBT. Note: one hydrogen bond per hydrogen atom is assigned at most in YASARA, thus the black arrow points to the missing hydrogen bond between Ser_105_ and His_224_.

CalB has limited space in the binding site pocket, giving rise to its selectivity [25]. By using the branched AB-type monomer as a substrate, the binding pocket of CalB becomes crowded. To evaluate the strained situation in the binding pocket, the homopolymerization of two MO-cys monomers was modelled by building the second tetrahedral intermediate, where one MO-cys makes a nucleophilic attack on the acyl-enzyme (CalB bound to one MO-cys). The simulations were performed in Yet Another Scientific Artificial Reality Application (YASARA) and gave a model for the binding position of the two MO-cys molecules in CalB. Snapshots of the modelling can be seen in Figure 3 and Figure 4.

In Figure 3a close up of the surface of the binding pocket is circled to the left and the homocondensation of two MO-cys monomers is shown to the right. The acyl donor MO-cys is shown in orange and the acyl acceptor is shown in blue. The model shows that the two MO-cys monomers are positioned without any steric clashes. Furthermore, during the molecular dynamics (MD) simulations, the important hydrogen bonds in the tetrahedral intermediate necessary for the catalytic activity of CalB were kept intact (Figure 4). 

The close up and schematic representation shown in Figure 4 display the important hydrogen bonds in the tetrahedral intermediate. As can be seen in the schematic representation and highlighted by the black arrow in Figure 4b, His_224_ forms hydrogen bonds with both the catalytic Ser_105_ and the acyl acceptor (blue). In the snapshot from the modelling shown in Figure 4a, one hydrogen bond is missing. This is due to the fact that only one hydrogen bond per hydrogen atom can be assigned in YASARA. The distance between the O in Ser_105_ and the H in His_224_ is 2.2 Å, i.e., within hydrogen bonding distance.

As can be seen in Table 1, the mole% of lipase was 1.2 × 10^3^ times less than for TBD. Lipase was added in 10 wt%, but typically contains a few wt% active lipases [21]. The high lipase activity can be attributed to the precise arrangements of functional groups constituting the amino acids, which enables the enzymes to bind to and position the reactant molecules so the transition-state is stabilized efficiently (see Figure 4) [25]. In contrast, the model for the organocatalyst provides less H-bonds in the stabilization of the transition state [16]. Thus, the possibility of lowering the activation energy is greater for the enzyme compared to the simple organocatalyst.

High conversions were reached for all reactions (Table 1), and the SEC analysis showed that high molecular weights were obtained. The main fractions in the SEC chromatograms for both catalysts showed *M*_n_ values around 7 × 10^3^ Da for reactions catalyzed at 80 °C and 18 × 10^3^ Da for the reactions catalyzed at 140 °C (supporting information Table S1).

All synthesized polymers were highly viscous at room temperature and no thermal transitions were observed by differential scanning calorimetry (DSC) analyses after cooling to −50 °C. The results are comparable with what was previously reported by Türünç et al. [15]. The melting point is largely affected by the density of amide groups [26] and thus these results were expected. 

MO-cys was copolymerized with 1,12-diaminododecane with the aim of synthesizing oligoamides with the degree of polymerization (DP) 2 or 4 at 80 °C (Scheme 2). The syntheses of both oligoamides were completed within 4 h (see results for Entry 5 and 6 in Table 1). 

**Figure 5:**
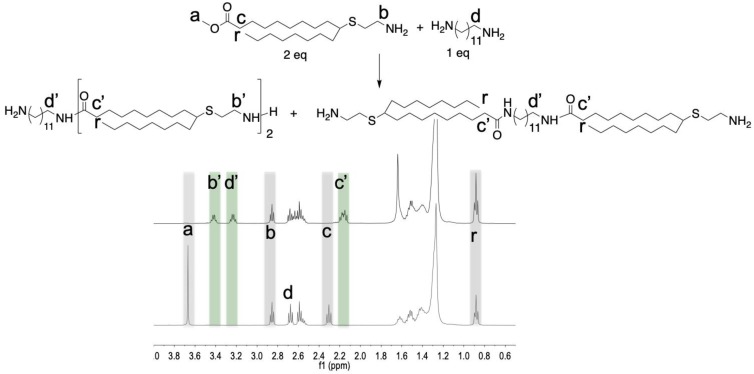
^1^H-NMR spectra (enlargements of the whole area), from 4.0–0.5 ppm of CalB catalyzed copolymerization of MO-cys and 1,12-diaminododecane at 80 °C. Samples were withdrawn after 4 h and diluted in CDCl_3_. Conversion was calculated from the formation of peak b’ and d’ using r as a reference. Signals highlighted in grey have been assigned to atoms present from the start (a, b, c and r) and signals highlighted in green have been assigned to atoms in the polymer (b’, c´ and r).

In Figure 5, the ^1^H-NMR analysis of the CaB catalyzed synthesis of the DP 2 oligoamide is shown. Full conversion of the methyl ester into oligoamide was achieved (peak c shifted to c’) and both amines participated as acyl acceptors in the amide formation (peaks d and b shifted to d’ and b’). The synthesis of the oligoamide with DP 4 showed that 1,12-diaminododecane was the preferred acyl acceptor for CalB and consequently 70% of the amine end groups came from MO-cys. 

## 3. Materials and Methods

Complete experimental description can be found in the supporting information.

### 3.1. Monomer Synthesis: Thiol-ene Addition of Cysteamine to Methyl Oleate

The procedure was adapted from original work published by Türünç et al. [15] and the synthesis described herein was performed with some modifications. Methyl oleate, cysteamine hydrochloride and 2,2-dimethoxy-2-phenylacetophenone (DMPA), in a molar ratio of 4:1:0.1, were weighed into a glass container and ethanol was added. The sample was irradiated with an intensity of approximately 25 mW cm^–2^ for 4 h at room temperature (22 °C). The reaction mixture was neutralized with a saturated Na_2_CO_3_ aqueous solution and extracted with diethyl ether twice. The reaction mixture was purified over silica gel, 10 g silica/1 gram sample. A mixture of hexane-ethyl acetate, in a 1:1 ratio, was used. To collect the final product, a mixture of methanol-ethyl acetate, in a 1:10 ratio was used. The collected product (MO-cys) was dried under vacuum and stored in the freezer to avoid dimerization.

### 3.2. General Procedure for Polymerizations 

All polymerization reactions were carried out in 5 mL round bottom flasks with continuous magnetic stirring and under vacuum (70 mbar) to drive the reaction to product formation by evaporation of formed methanol. The reactions were monitored by ^1^H-NMR. 

**Copolymerization**: The monomer synthesized by radical thiol-ene addition, MO-cys, was copolymerized with 1,12-diaminododecane at 80 °C, using CalB as a biocatalyst, in a molar ratio of MO-cys: 1,12-diaminododecane (4:1) for DP4 and (2:1) for DP2. The reaction was run in bulk and started by addition of 10 wt% of CalB.

**Homopolymerization**: Reactions were run in bulk at 80 °C and 140 °C. MO-cys (200 mg) was added using either CalB (10 wt%) or TBD (0.05 equivalents to the monomer) as catalysts.

Conversion and degree of polymerization (DP) were determined by ^1^H-NMR. Conversion was calculated from the formation of peak b’ (homopolymerization) or peak b’ and d’ (copolymerization) with peak r as reference. The degree of polymerization (DP) was calculated from peak b (homopolymerization) or peak b and d (copolymerization) in relation to peak r.

## 4. Conclusions

Immobilized *Candida antarctica* lipase B (CalB) could be used as an efficient catalyst to produce renewable polyamides of high molecular weights and amine end-capped oligoamides. The experimental work demonstrated that CalB works efficiently although modelling showed a sterically strained situation. When aiming for polymers with higher molecular weights, the temperature should be raised, or solvent should be introduced in order to overcome viscosity limitations. An additional synthesis advantage of using immobilized CalB as a heterogeneous catalyst is that this catalyst can easily be removed from the final product mixture while the TBD catalyst is left in the product. At 80 °C CalB catalyzed the synthesis of oligoamides within 3.5 h in solvent-free reaction conditions. The oligoamides can be further used as one of the building blocks in the synthesis of block copolymers.

## Figures and Tables

**Table 1:** Polycondensation of MO-cys: homopolymerization or copolymerization with 1,12-diaminododecane (DA).

Entry	Catalyst	mol% Catalyst ^a^	Ratio MO-cys:DA	Temp (°C)	Time (h)	Conversion ^b^ (%)	DP ^b^
1	CalB	0.004	1:0	80	20	>85	>6
2	CalB	0.004	1:0	140	20	>95	>20
3	TBD	5	1:0	80	20	>85	>6
4	TBD	5	1:0	140	20	>95	>20
5	CalB	0.004	2:1	80	4	>98	2
6	CalB	0.004	4:1	80	4	>98	4
7	- ^c^	-	1:0	80	20	20	-
8	- ^c^	-	1:0	140	20	30	-

**^a^** Assuming 3.3 wt% active lipase immobilized on carriers [21].^**b**^ Determined by ^1^H-NMR. **^c^** Control reactions without catalyst.

## Supplementary Materials

The following are available online at https://www.mdpi.com/2073-4360/11/11/1730/s1, Complete experimental description, Figure S1. ^1^H-NMR of cysteamine coupled to methyl oleate, after purification. Figures S2–S6 and Table S1 THF GPC results.
