# Supplementary material for: Lipase-Catalyzed Synthesis of Renewable Plant Oil-Based Polyamides
Source: Polymers (Basel). 2019 Oct 23;11(11):1730. doi: 10.3390/polym11111730 (PMC6918247; doi:10.3390/polym11111730)
Supplement: Supplementary file 1 [file polymers-11-01730-s001.pdf]

polymers

Lipase Catalyzed Synthesis of renewable plant oil-based polyamides

Maja Finnveden [1], Peter Hendil-Forssell [1], Mauro Claudino [2], Mats Johansson [2] and Mats Martinelle [1*]

[1] Department of Industrial Biotechnology, KTH Royal Institute of Technology, SE-106 91 Stockholm, Sweden
[2] Department of Fibre and Polymer Technology, KTH Royal Institute of Technology, SE-100 44 Stockholm, Sweden
* Correspondence: matsm@kth.se

Materials

All chemicals were purchased from Sigma-Aldrich and used as received unless noted. Novozyme 435 (*Candida antarctica* lipase B immobilized on acrylic carrier, ≥5000 U g^{-1}) was stored in a desiccator with LiCl. Silica gel pore size 60 Å (particle size 35–75 μm).

Monomer synthesis - Thiol-ene addition of cysteamine to methyl oleate

Methyl oleate (10 g, 33 mmol), cysteamine hydrochloride (1 g, 8.6 mmol), and 2,2-dimethoxy-2-phenylacetophenone (DMPA) (2 wt %, 220 mg, 0.86 mmol), in a molar ratio of (4 : 1 : 0.1), were weighed into a glass container and 8 mL of ethanol was added. The mixture was magnetically stirred and irradiated with a UV lamp at room temperature (22°C). The sample was irradiated with an intensity of approximately 25 mW cm^{-2} for 4 h (dose 360 J cm^{-2}). The intensity was determined with an UVICURE Plus High Energy UV integrating radiometer (EIT, USA), measuring UVA at $320 \leq \lambda \leq 390$ nm. The crude product was a viscous yellowish oil. The reaction mixture was neutralized with a saturated Na_2CO_3 aqueous solution and extracted with diethyl ether twice. The product was dried under vacuum. To remove all unreacted residue (unsaturated methyl oleate) the reaction mixture was purified over silica gel, 10 g silica was used per 1 gram of loaded sample. A mixture of hexane-ethyl acetate, in a 1:1 ratio, was used. To collect the final product, a mixture of methanol-ethyl acetate, in a 1:10 ratio was used. The collected product (MO-cys) was still yellowish. MO-cys was dried under vacuum and stored in the freezer to avoid dimerization due to disulfide formation.

The conversion of methyl oleate to MO-cys was 80%. The conversion was calculated from the ^{1}H-NMR analysis, from the decrease of the peak corresponding to the double bond of methyl oleate at 5.38 ppm. Moreover, the conversion from methyl oleate to MO-cys was evidenced by the formation of new peaks at 2.5 ppm and 2.59 ppm, corresponding to the protons of the α- position of the thioether on the fatty acid chain. HMBC was used to properly assign the ^{1}H-NMR. The reactions mixture was purified over a silica gel column and the final ^{1}H-NMR (see Figure S 1) analysis of the synthesized monomer showed a pure spectrum. The structure of MO-cys was further confirmed by ^{13}C-NMR.

^{13}C NMR (400 MHz, CHCl3-d, δ in ppm) δ = 174.24 (-COO-), δ = 51.42 (-COO-CH3), δ = 45.87 (>CH-S-), δ = 41.71 (NH2-CH2-), δ = 35.01 (-S-CH2 -), δ = 34.71 (-CH2-COO-), δ = 35.09-24.91 (aliphatic), δ = 14.12 (-CH2- CH3)

General procedure for polymerizations

All polymerization reactions were carried out in 5 mL round bottom flasks with continuous magnetic stirring and vacuum (70 mbar) to evaporate formed methanol and drive the reaction towards completion. The reactions were monitored by ^1H-NMR. Filtering off the enzyme carriers using a cotton filter stopped reactions catalyzed by CalB and reactions catalyzed by TBD were stopped by immediately freezing the sample with liquid nitrogen.

Copolymerization: MO-cys was copolymerized with 1,12-daminododecane at 80°C, using CalB as a catalyst. The reaction was run for 4 h in bulk and was started by the addition of 10 wt% CalB.

DP2: MO-cys (290 mg, 80 mmol) and 1,12-diaminododecane (80 mg, 40 mmol) in a molar ratio of MO-cys: 1,12-diaminododecane (2:1)

DP4: MO-cys (290 mg, 80 mmol) and 1,12-diaminododecane (40 mg, 20 mmol) in a molar ratio of MO-cys: 1,12-diaminododecane (4:1)

Homopolymerization: MO-cys was homopolymerized in 200 mg scale and started by the addition of CalB (10 wt% relative to the total weight of the monomer) or TBD (0.05 equivalents to the monomer). The reactions were run for 20 h at 80 and 140°C. Additionally reactions without catalyst were run for 20 h at 80 and 140°C

Analytical methods

Thin layer chromatography (TLC) was performed on silica gel TLC-cards, compounds visualized by ultraviolet light (254 nm).

Nuclear magnetic resonance (NMR) both, ^1H-NMR, ^{13}C-NMR and HMBC spectra were recorded on a Bruker AM 400. Deuterated $CDCl_3$ containing 1 vol% tetramethylsilane (TMS) was used as solvent. The ^1H-NMR spectra were based on 16-40 scans, ^{13}C-NMR spectra on 4096 scans.

Size-exclusion chromatography (SEC) was performed on a Malvern VISCOTEK GPCmax equipped with a refractive index detector and TGuard column followed by two linear mixed bed columns (LT4000L) (35°C). Tetrahydrofuran (THF) stabilized with butylated hydroxytoluene (BHT) was used as mobile phase (1 mL/min). A calibration method was created using narrow linear polystyrene standards. All samples were filtered through a 0.2 μm PTFE filter (13 mm, PP housing, Alltech) prior to analysis.

Differential scanning calorimetry (DSC). Thermal analyses were carried out on a Mettler Toledo DSC-1 equipped with Gas Controller GC100. For the analysis 5–10 mg of each sample was weighed into aluminum crucibles of 100 μl. The data was collected using a heating/cooling rate of 5°C min^{-1} from -60°C to 150°C and 5 min isotherms with a nitrogen gas atmosphere of 30 ml min^{-1}. The glass transition temperature (T_g) was obtained as the midpoint of the heat capacity change and was taken from the second heating scan. Evaluation of acquired data was done using STARe Excellence Software.

Molecular modelling

Molecular dynamics (MD) simulations were performed in Yet Another Scientific Artificial Reality Application (YASARA) version 18.12.27 using PDB entry 1LBT. The Amber14 force field was used with AutoSMILES approach for force field parameter assignment, Particle Mesh Ewald accounted for long-range electrostatic interactions and a cut-off of 10.5 Å for van der Waals interactions. The crystal structure was processed by building the second tetrahedral intermediate from the inhibitor that binds to the catalytic serine (Ser$_{105}$) and by adding missing hydrogens. The two MO-cys monomers, acyl donor and acyl acceptor were sequentially built to resemble a tetrahedral intermediate during deacylation. Energy minimizations and MD runs (0.005 ns) were made for each added carbon. After the tetrahedral intermediate was built MD runs for 0.02ns followed by energy minimizations was done.

Results

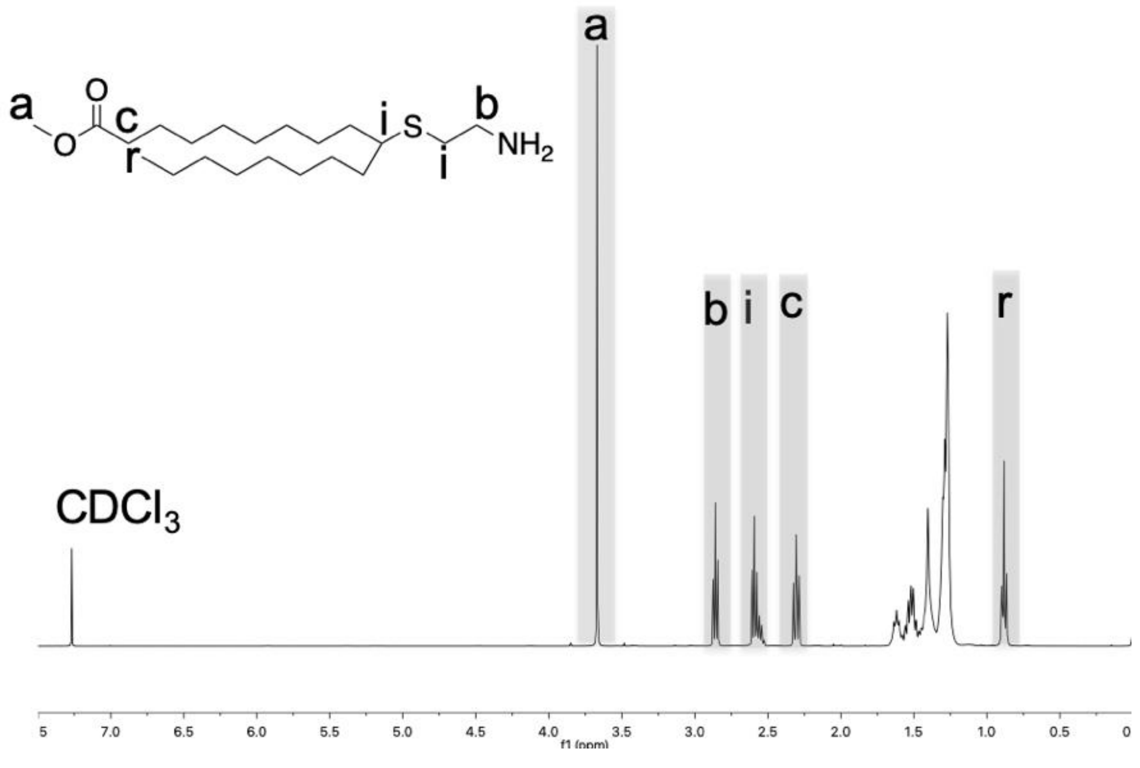

Figure S 1. ^{1}H-NMR of cysteamine coupled to methyl oleate, after purification.

SEC

THF SEC chromatograms of all polycondensation products are included below. All products contained traces of low molecular weight compounds. The selected regions discussed in the communication are thus to be looked at with caution. However, the data is used to indicate the M_n of the main fraction. In addition to M_n, the peak molecular weight (M_P) is given in Table S 1. M_P is the molecular weight of the highest peak.

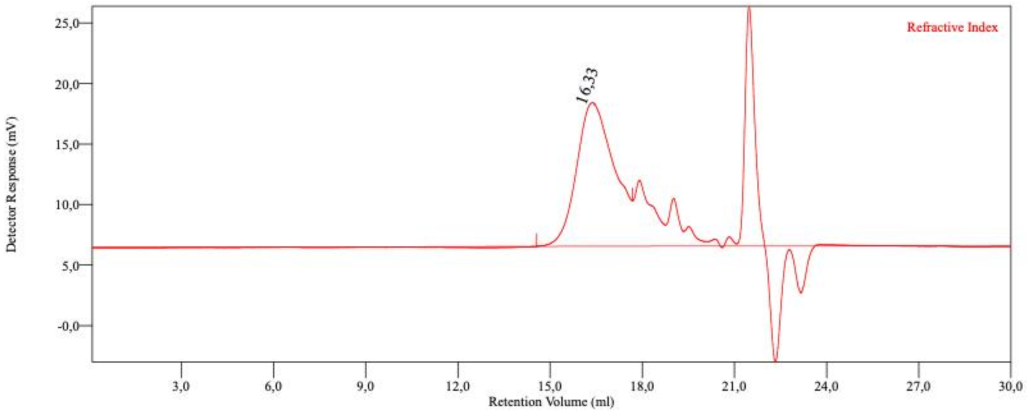

Figure S 2. THF SEC of DP4 oligoamide.

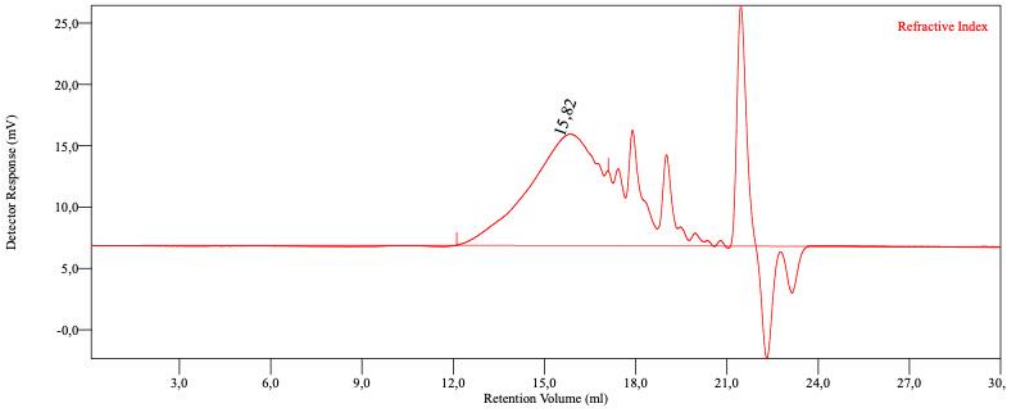

Figure S 3. THF SEC of polymer from CalB-catalyzed homopolymerization of MO-cys at 80°C

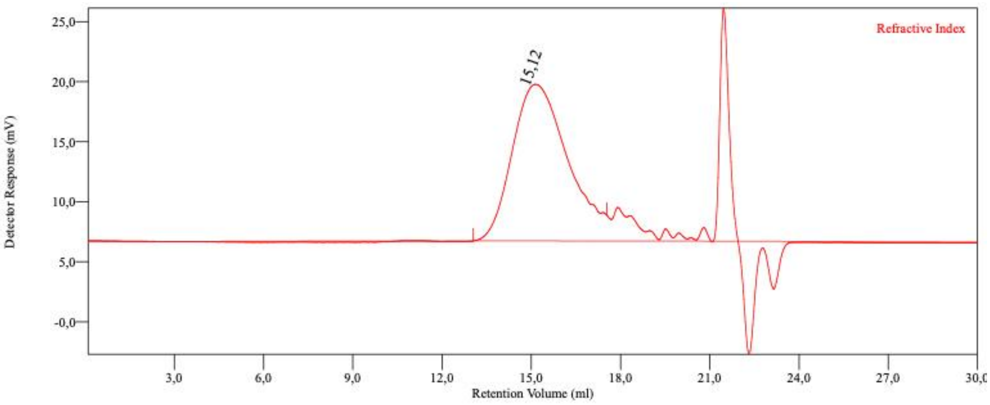

Figure S 4. THF SEC of polymer from TBD-catalyzed homopolymerization of MO-cys at 80°C

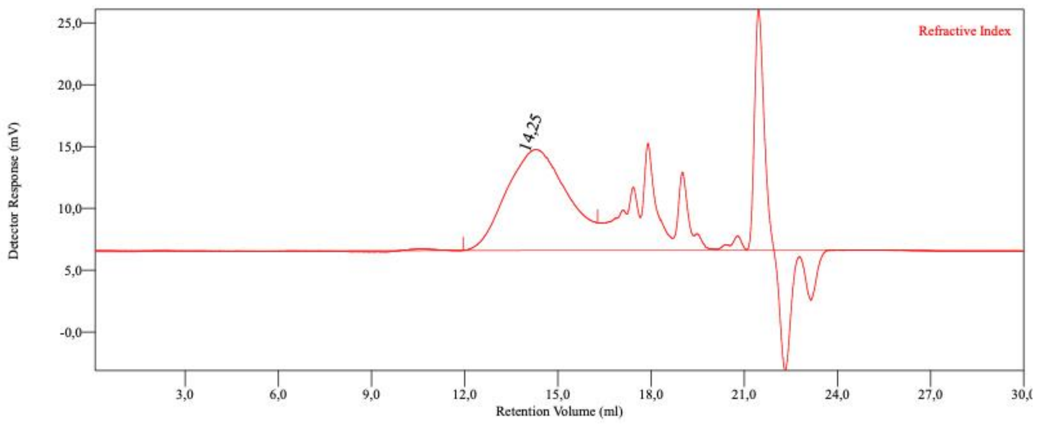

Figure S 5. THF SEC of polymer from CalB-catalyzed homopolymerization of MO-cys at 140°C

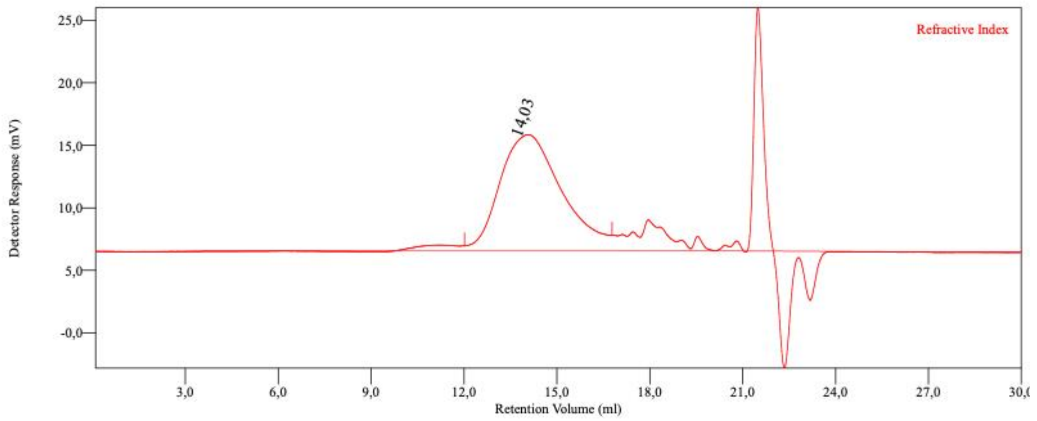

Figure S 6. THF SEC of polymer from TBD-catalyzed homopolymerization of MO-cys at 140°C

Table S 1. M_n and M_p data from polyamide synthesis

**Entry**	**Catalyst**	**Ratio MO-cys:DA**	**Temp (℃)**	**Time (h)**	**M_n [a] (g/mol)**	**M_p (g/mol)**
1	CalB	1:0	80	20	6700	6600
2	CalB	1:0	140	20	18000	25000
3	TBD	1:0	80	20	7200	12000
4	TBD	1:0	140	20	18000	30000
5	CalB	2:1	80	4	-	-
6	CalB	4:1	80	4	3200	4300

[a] From the main region (see marks in chromatogram Figure S 2-6).
